# Achieving coronal plane alignment in total knee arthroplasty through modified preoperative planning based on long-leg radiographs: a prospective study

**DOI:** 10.1186/s40634-021-00418-y

**Published:** 2021-11-02

**Authors:** Daria Singh, Kalpeshkumar C. Patel, Ragini D. Singh

**Affiliations:** 1 Zydus Hospitals, Ahmedabad, Gujarat 380059 India; 2 Department of Biochemistry, All India Institute of Medical Sciences, Khanderi, Rajkot, Gujarat India

**Keywords:** Conventional TKA, Preoperative planning, True-alignment technique

## Abstract

**Purpose:**

This prospective study was undertaken to examine whether the desired coronal plane alignment of limb and prosthetic components in total knee arthroplasty (TKA) could be achieved precisely using conventional jig-based methods by modifying the preoperative planning of bone resection utilizing long-leg radiographs (LLRs).

**Methods:**

The study included consecutive 245 TKA procedures. Pre- and postoperative radiological variables, i.e., the mechanical axis (hip-knee-ankle [HKA] axis), mechanical lateral distal femoral angle (mLDFA), and medial proximal tibial angle (MPTA), and their outliers were evaluated. Statistical analysis was performed using SPSS version 21.0.

**Results:**

The mean postoperative HKA axis, mLDFA and MPTA was 179.80 ± 1.81° (*p* < 0.01; 95% CI: 8.09–9.67), 90.35 ± 1.54° (*p* < 0.01; 95% CI: 1.33–2.02), and 90.26 ± 1.25° (*p* < 0.01; 95% CI: 4.41–5.20), respectively. The postoperative HKA axis on the coronal plane was 180 ± 3° in 235 knees (95.92%, 4.08% outliers). Femoral and tibial components were implanted in an acceptable position, withing 90 ± 3° of the mechanical axis of the femur and tibia on the coronal plane in 238 (97.14%, 2.86% outliers) and 243 (99.18%, 0.8% outliers) knees, respectively.

**Conclusion:**

Modified preoperative planning for TKA on LLRs is a reliable and consistent method to achieve the desired limb and component alignment on the coronal plane without adding financial or logistical costs.

**Level of evidence:**

II

## Background

Total knee arthroplasty (TKA) is a reliable surgical intervention to treat pain and disability caused by advanced arthritis of the knee. The utilization of TKA is increasing at a fast pace worldwide, owing to the increasing aging population and improved outcomes of TKA. The demand for TKA is also increasing in young, active patients with knee arthritis who expect a better functional outcome and longer implant survival [35]. Due to the large absolute number of TKA procedures being performed, even a small percentage of error can lead to a very large absolute number of TKA procedures being at risk for poor functional outcomes, patient dissatisfaction, and early implant failure. Neutral mechanical alignment (180 ± 3°) on the coronal plane is one of the most important determinants of successful TKA [1]. Many published studies have suggested that TKA with good alignment on the coronal plane leads to a better functional outcome and enhanced patient satisfaction [10, 16, 23]. The neutral mechanical axis reduces contact stresses on the implant and its subsequent wear [22, 39] and related failures, and stresses on the bone-implant interface and thereby enhances implant survival [14, 40]. A frequently cited study by Parratte et al. observed that better alignment did not lead to increased implant survival. However, the authors also concluded that until additional data can be generated to determine the ideal postoperative alignment in an individual patient, the neutral mechanical axis remains a reasonable target [30].

There are various alignment options, including anatomical, anatomical-like, mechanical, adjusted mechanical, kinematic, and restricted kinematic alignment, to recreate a mechanical environment conducive to a better functional outcome and increased implant survival [33]. However, an overwhelmingly large number of studies support the concept of mechanical alignment [1]. There are various surgical techniques, such as conventional surgery, optimized conventional surgery [26], computer-assisted surgery (CAS) [4], patient-specific instrumentation (PSI) [6], and robot-assisted surgery [8], to achieve the target limb and component alignment according to the alignment option adopted. Despite technological advances and improvements in understanding, achieving neutral mechanical alignment on the coronal plane remains a daunting task. In conventional jig-based methods, the rate of achieving neutral alignment is abysmally low because of large anatomical variabilities [34, 38], a fixed valgus cutting angle (VCA) [12, 27], aberrant jig placement [28], intraoperative cutting errors [7], the lack of a consensus on the proximal tibial landmark for fixing tibial jigs [2, 11, 15] and the inherent limitations of jigs [9, 32]. Various technologies, such as CAS, PSI, and robot-assisted TKA, have been developed in an effort to improve the rate of achieving neutral mechanical alignment, but all of these methods included additional variable costs and the potential for complications unique to the technology used [37]. These technologies are increasingly being used in the developed world. However, the affordability and availability of these technologies are still problems in developing countries, where increasingly large numbers of TKA procedures are being done with conventional methods [3, 31].

Therefore, the present study aimed to determine whether a modified preoperative planning method (i.e., the true-alignment technique) based on long-leg radiographs (LLRs) can consistently achieve neutral mechanical alignment of the leg as well as alignment of the femoral and tibial components in TKA using conventional jigs while not incurring any additional cost or increasing the risk of complications. Given current knowledge, the hypothesis was that a neutral mechanical limb axis and femoral and tibial component alignment on the coronal plane could be consistently achieved with the method described in the present paper.

## Material and methods

### Study design

This noncomparative, open-label, single-arm clinical study was carried out after ethics committee approval was obtained. The sample size was calculated using an equality test for a one-sample design (power of 80%, α of 0.05 and standard deviation of 1.7 for the mechanical axis) [26]. Based on these estimates, a total of 235 cases were required. Considering a ~ 5% dropout and/or withdrawal rate, *N* = 245 knees were included. The inclusion criteria comprised informed consent, coronal plane deformity < 25° varus or valgus, sagittal plane deformity < 15°, and satisfactory LLRs. Knees with > 25° coronal plane deformity, > 15° sagittal plane deformity, or unsatisfactory LLRs were excluded. We screened 239 consecutive patients who underwent TKA between October 2018 and March 2019 and included 191 patients (245 knees) and excluded 48 patients (48 knees). Twenty-three knees with coronal plane deformity > 25°, 13 knees with sagittal plane deformity > 15° and 8 knees with poor-quality LLRs (nonvisualization of the center of the femoral head) were among the excluded knees. Four knees were excluded due to nonavailability of LLRs due to X-ray machine breakdown. No patients were excluded or dropped out postoperatively.

### Surgical planning

All surgeries were planned and performed by the first author (DS). As a reliable tool for measuring the mechanical axis and component position, standing LLRs were used to measure all pre- and postoperative radiological parameters and plan proximal tibial and distal femoral bone resection [13]. Standardized digital LLRs were obtained in the standing position by a previously described method [13]. Radiographs were evaluated for acceptable image quality and rotation by assessing the profile of the lesser trochanter, the positioning of the patella over the femur (central position was accepted), and the amount of overlap of the fibular head on the tibia (one-third of fibular head overlap was accepted). The radiographs were considered satisfactory when the above criteria for at least two landmarks were met. Once satisfactory radiographs were obtained, the patients were included in the study. Postoperative standing LLRs were obtained when patients were able to complete a full active straight-leg raise and stand straight on the radiography platform. The following pre- and postoperative coronal plane radiological parameters were measured on a digital radiograph workstation: (i) the mechanical axis (hip-knee-ankle [HKA] axis), i.e., the angle between the femoral mechanical axis (line joining the center of the femoral head and the center of the femoral/femoral implant trochlear notch), and the tibial mechanical axis, i.e., the line joining the point between the tibial spine/center point of the tibial base plate to the central depression on the dome of the talus; (ii) the mechanical lateral distal femoral angle (mLDFA), i.e., the lateral angle between the femoral mechanical axis and the tangent line connecting the distal femoral condyle preoperative and femoral prosthesis postoperative; and (iii) the medial proximal tibial angle (MPTA), i.e., the medial angle between the tibial mechanical axis and the tangent line connecting the proximal tibial condyles preoperative and tibial component postoperative. The aim was to restore the mechanical axis classically within 3° of the neutral mechanical axis (HKA axis 180 ± 3°).

The second author (KCP) measured and recorded all the pre- and postoperative outcome variables. Since he was not the operating surgeon, the use of this method also helped to avoid any operator bias. The outcome variables of 20 randomly selected knees were remeasured after 2 weeks by KCP. These variables in the same set of knees were also measured by DS to test the intraobserver and interobserver variability.

### Distal femoral resection planning

**Fig. 1:**
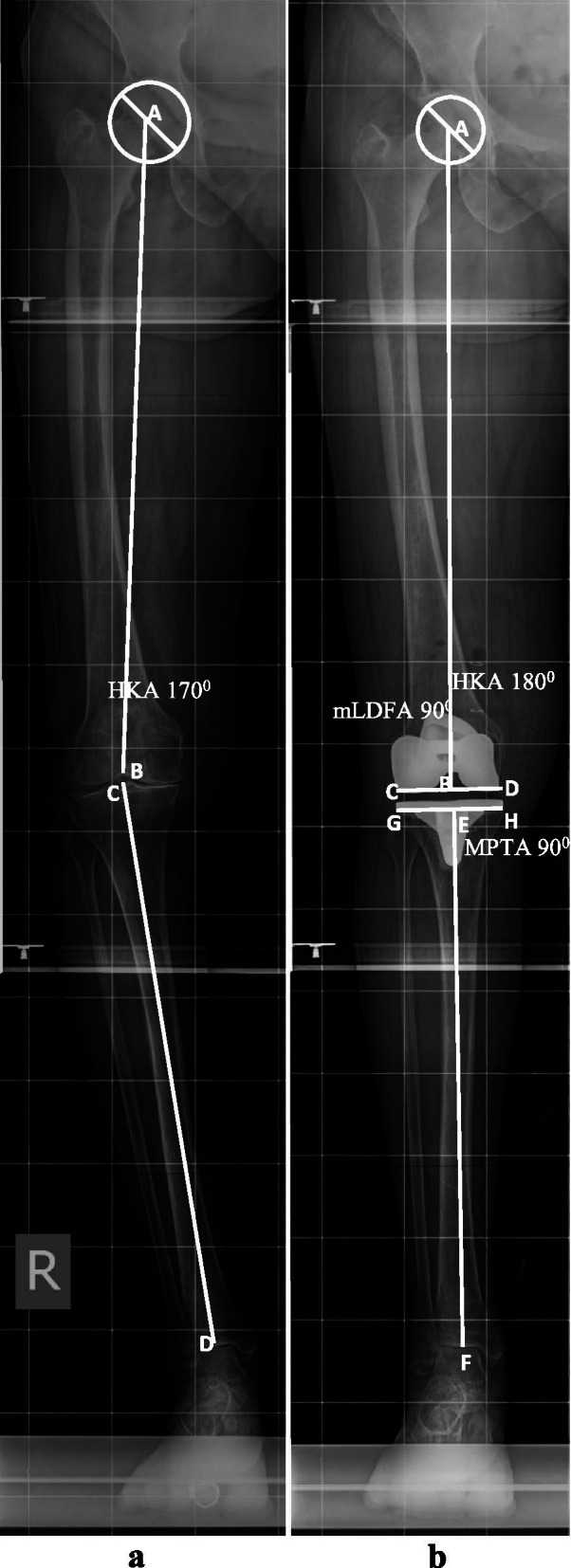
**a** Preoperative standing LLR denoting the centre of femoral head (point A), centre of the distal femoral articular surface (point B), femoral mechanical axis (Line AB), centre of the proximal tibia/centre of tibial spine (point C), centre of the distal tibial articular surface, coinciding with midtalar groove (point D), HKA angle i.e. angle between line AB and CD is 168**°** or 12**°** varus. **b** Postoperative standing LLR denoting HKA angle, (angle between line AB and EF) 180**°**, mLDFA, angle ABC, 90**°** and MPTA, angle FEH, 90**°**

**Fig. 2:**
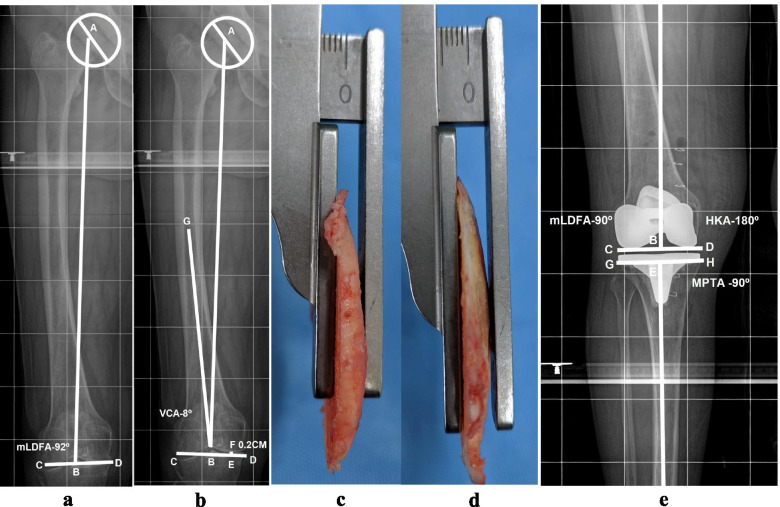
**a** Depicting the mechanical axis of femur (Line AB), and a tangential line to the distal femoral articular surface (Line CD) and preoperative mLDFA (angle ABC), 92**°** that means 2**°** varus correction in femur is required. **b** Depicting the femoral mechanical axis (Line AB), and a line perpendicular to mechanical axis, a line tangential to at least one distal femoral condyle, and in this case lateral distal femoral condyle, (Line CD). Line CD is 0.2 cms away from medial femoral condyle, meaning bone cut from medial femoral condyle must be 0.2 cm less than lateral femoral condyle. Line GB is trajectory for intramedullary road and this line makes 82**°** angle (angle GBC), which means VCA of 8**°**. **c** Intra-operative photograph showing thickness of the bone resected from the medial femoral condyle, 4.5 mm. **d** Intra-operative photograph showing the thickness of the bone resected from the lateral femoral condyle, 6 mm (planned difference of thickness of bone resection from medial and lateral femoral condyle was 2 mm, here we could get 1.5 mm difference). **e** Post-operative LLR (showing only knee). Depicting the mLDFA, angle ABC, 90**°** (planned femoral component position)

The aim of distal femoral resection was to cut the distal femur at 90° to its mechanical axis. The femoral mechanical axis (line AB) joining the center of the femoral head (point A) and the deepest point of the intercondylar notch (point B) on LLRs (Figs. 1a & 2a) was drawn. Then, a second line (CD) was drawn tangent to the distal femoral articular surface. This angle ABC was the mLDFA (Fig. 2a). If the mLDFA was 90°, then bone cuts of equal thickness for both distal femoral condyles were planned. If the mLDFA was > 90° or < 90°, then another line (CD) perpendicular to the mechanical axis of the femur (line AB) and tangent to at least one femoral condyle was drawn. The length of line EF was the difference in bone resection between the two condyles (Fig. 2b). Since intramedullary jigs were used to guide resection, the VCA were calculated by drawing a line from the deepest point of the trochlear groove (portal of entry) to the center of the medullary canal at 23 cm from the portal of entry (line BG). Line BG was the trajectory for the intramedullary rod, and angle GBC was the planned VCA (pVCA) (Fig. 2b). Intraoperatively, bone to be resected from the distal femoral condyle with the pVCA was measured after removing cartilage from the unaffected condyle. If it matched the preoperative plan for bone resection, then the same bone thickness was resected. If it did not match, then the VCA was adjusted to the planned bone resection thickness. The final VCA read on the jig was the executed VCA (eVCA). The femoral component position was confirmed on postoperative LLRs (Fig. 2c, d & e).

### Proximal tibial resection planning

**Fig. 3:**
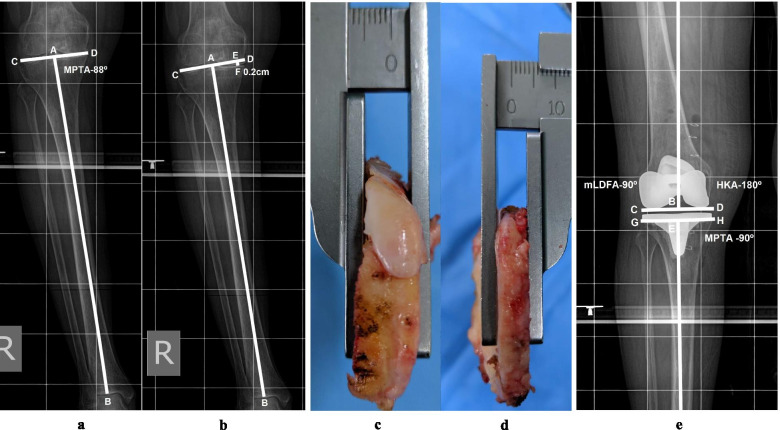
**a** LLR (showing only tibia). Point A - a point in the middle of both tibial spine, point B coinciding with depression on dome of talus, line AB - tibial mechanical axis, line CD - A tangential line to the both proximal tibial condyles, Angle ABD is MPTA (88**°**), meaning proximal tibia 2**°** varus. **b** Drawing of planned proximal tibial resection, line AB mechanical axis, line CD is perpendicular to the mechanical axis passing through point A and tangential to unaffected proximal tibial condyle, here in this case lateral tibial condyle. Line EF perpendicular to Line CD and point F is deepest point at margin of medial tibial condyle, length of this line denotes difference between planned thickness of proximal tibial resection, here planned tibial resection was 2 mm more from lateral tibial condyle than medial tibial condyle after removing cartilage from lateral tibial condyle (with cartilage, depth of resection was planned 9 mm from unaffected condyle, lateral condyle in this case). **c** Intra-operative measurement of thickness of the resected proximal lateral tibia, 8 mm (2 mm more than medial proximal tibial condyle). **d** Intra-operative measurement of thickness of the resected proximal medial tibia, 6 mm. **e** Postoperative LLR (showing only knee) illustrating MPTA, angle FEH, 90**°**, planned tibial component position

Proximal tibial resection was planned at 90° to the tibial mechanical axis. The tibial mechanical axis (line AB) was drawn using the center of the tibial spine (point A) and the deepest point of the superior articular surface of the talus (point B) (Fig. 3a). The second line CD was drawn tangent to both proximal tibial condyles. The angle BAD was the MPTA. Then, a line (CD) tangent to the unaffected proximal condyle and perpendicular to the tibial mechanical axis (AB) was drawn, and the difference in the thickness of the bone to be resected was measured, i.e., the length of line EF (Fig. 3b). Intraoperatively, the mark on the superior surface of the cutting block of the extramedullary tibial jig was aligned to a line drawn on the center of the tibial spine from anterior to posterior ignoring the position of the tibial tubercle. On the unaffected side, the planned depth of resection required for the thinnest tibial component was measured, with a minimum of 9 mm for the ATTUNE Knee System (Johnson and Johnson), including bone and cartilage. Then, cartilage from the unaffected proximal tibial condyle was removed, and the thickness of bone to be resected (9 mm minus the cartilage thickness) was remeasured and matched to the preoperative plan. Similarly, the thickness of bone to be resected on the affected side was measured and matched to that in the preoperative plan. If there was any discrepancy, then the jig was adjusted accordingly. The thickness of the resected bone was measured and matched to that in the preoperative plan and confirmed on postoperative LLRs (Fig. 3c, d & e).

The procedures were performed under combined spinal-epidural or spinal anesthesia. General anesthesia was given when spinal and epidural anesthesia were contraindicated. All procedures were performed using a tourniquet except in patients with heavily calcified arteries in the lower limbs. The ATTUNE Knee System (J&J) was implanted using parapatellar arthrotomy. Routine perioperative antibiotics and thromboprophylaxis (enoxaparin) were administered for 48 h and 5 days, respectively.

### Outcome measures

All demographic data and outcome variables were measured and recorded prospectively by KCP. The radiological variables (outcome variables) recorded were the pre- and postoperative mechanical axis (HKA axis), mLDFA, and MPTA. Other radiographic measures recorded were the pVCA and eVCA.

### Statistical analysis

The data were analyzed using SPSS statistical software (version 21.0, IBM Corp., Armonk, N.Y., USA). Determination of the change in the outcome parameters between preoperatively and postoperatively was carried out using a paired -sample t-test. Correlations between the HKA axis, mLDFA, and MPTA were assessed using Pearson’s correlation coefficient. The intraobserver and interobserver variability were also evaluated. Fischer’s exact test was applied to compare the percentage of outliers in the radiological parameters between groups stratified by age, sex, side, body mass index (BMI), and type and severity of deformity. *P* values ≤0.05 were considered to indicate statistical significance.

## Results

There were 245 knees in 191 patients, including 147 (76.96%) females and 44 (23.03%) males, with a mean age of 63.93 ± 7.29 years. A total of 56 patients (112 knees) underwent TKA on both sides simultaneously; of these, two knees in two patients were excluded because the preoperative coronal plane deformity was more than 25° (110 knees were included from the bilateral group), while 135 patients (135 knees) underwent TKA on one side. Other demographic details and patient characteristics are provided in Table 1.

**Table 1:** Demographic details of the study cohort (*N* = 245)

**Age (years)**	Mean ± SD = 63.93 ± 7.29 Range: 46.0–84.0 95% CI: 62.89–64.99
**Sex**	
Male	44 (23.04%)
Female	147 (76.96%)
**BMI (kg/m**^**2**^**)**	Mean ± SD = 30.27 ± 4.81 Range: 22.0–49.5 95% CI: 29.27–30.76
**Side**	
Left	117 (47.75%)
Right	128 (52.25%)
**Unilateral**	135 knees (55.1%)
**Bilateral**	110 knees (44.9%) in 56 patients^a^
**Deformity**	
Varus	222 (90.61%)
Valgus	18 (7.34%)
Neutral	05 (2.04%)

^a^One limb each in two patients had coronal plane deformity more than 25^0^ leaving a total number of 110 knees, in the bilateral group

### Outcome variables

#### HKA axis

Postoperatively, the HKA axis was restored to a mean of 179.80 ± 1.81° from a preoperative mean of 170.91° ± 6.56° (*p* < 0.01; 95% CI: 8.09–9.67) (Table 2). The HKA axis was within 180 ± 3° in 235 knees (95.92%) and outside this range in 10 knees (outliers, 4.08%). All the outliers were in the preoperative varus deformity group (10/227, 4.40%). These outliers were further stratified according to the severity of deformity, as follows: ≤15° in 7/193 (3.62%) and > 15° in 3/34 (8.82%) (Table 3).

**Table 2:** Pre- and postoperative primary outcome variables (Radiographic results)

Radiographic variable	Preoperative Mean ± SD and Range 95% CI	Postoperative Mean ± SD and Range 95% CI	***p*** value; 95% CI
**HKA axis (**^**0**^**)**	170.91 ± 6.56 (Range: 156–199) 95% CI: 170.08–171.74	179.80 ± 1.81 (Range: 174–185) 95% CI: 179.56–180.02	< 0.01; 8.09–9.67
**mLDFA (**^**0**^**)**	88.68 ± 2.59 (Range: 81–96) 95% CI: 88.35–89.00	90.35 ± 1.54 (Range: 86–95) 95% CI: 90.16–90.55	< 0.01; 1.33–2.02
**MPTA (**^**0**^**)**	85.41 ± 3.38 (Range:76–102) 95% CI: 84.97–85.82	90.26 ± 1.25 (Range: 86–94) 95% CI: 90.09–90.41	< 0.01; 4.41–5.20

**Table 3:** Comparison of outliers in primary variables in groups stratified by age, sex, side, BMI and type and severity of the deformity

Radiographic variable			
**Age**	**≤65 years,** *N* = 144	**> 65 years,** *N* = 101	***p*** **value**
HKA axis, Outlier (%)	06, (4.16%)	04, (3.96%)	1.00
mLDFA, Outlier (%)	05, (3.47%)	02, (1.98%)	0.70
MPTA, Outlier (%)	01, (0.69%)	01, (0.99%)	1.00
**Sex**	**Male,** *N* = 52	**Female,** *N* = 193	
HKA axis, Outlier (%)	01, (1.92%)	09, (4.66%)	0.69
mLDFA, Outlier (%)	00, (0.00%)	07, (3.62%)	0.35
MPTA, Outlier (%)	01, (1.92%)	01, (0.51%)	0.38
**Side**	**Left,** *N* = 116	**Right,** *N* = 129	
HKA axis, Outlier (%)	05, (4.31%)	05, (3.87%)	1.00
mLDFA, Outlier (%)	03, (2.58%)	01, (0.007%)	0.34
MPTA, Outlier (%)	01, (0.86%)	04, (3.10%)	0.37
**BMI**	**≤30 kg/m**^**2**^**,** *N* = 124	**> 30 kg/m**^**2**^**,** *N* = 121	
HKA axis, Outlier (%)	04, (3.22%)	06, (4.95%)	0.53
mLDFA, Outlier (%)	03, (2.41%)	04, (3.30%)	0.71
MPTA, Outlier (%)	01, (0.80%)	01, (0.83%)	1.00
**Type severity of the deformity**	**Varus,** *N* = 227	**Valgus,** *N* = 18	
HKA axis, Outlier (%)	10, (4.40%)	00, (0.00%)	0.61
mLDFA, Outlier (%)	07, (3.08%)	00, (0.00%)	1.00
MPTA, Outlier (%)	02, (0.88%)	00, (0.00%)	1.00
**Severity of the varus deformity**	**≤15**^**0**^, *N* = 193	**> 15**^**0**^, *N* = 34	
HKA axis, Outlier (%)	07, (3.62%)	03, (8.82%)	0.17
mLDFA, Outlier (%)	06, (3.10%)	01, (2.94%)	1.00
MPTA, Outlier (%)	01, (0.51%)	01, (2.94%)	0.27

#### Femoral and tibial component coronal plane alignment

Femoral components were implanted at a mean mLDFA of 90.35 ± 1.54°. The mean preoperative mLDFA was 88.68 ± 2.59° (*p* < 0.001; 95% CI: 1.33–2.02) (Table 2). The mean pVCA was 5.92 ± 1.08° (range: 3–9°), and the mean eVCA was 5.91 ± 1.15° (range: 3–10°). A total of 238 femoral components (97.14%) were implanted within 90 ± 3° of the mLDFA. The eVCA was different from the conventional range of 5–7° in 32/245 (13.06%) knees and different from the pVCA in 20 (8.16%) knees. Tibial components were implanted at a mean of 90.26 ± 1.25°. The mean preoperative MPTA was 85.41 ± 3.38° (*p* < 0.01; 95% CI: 4.41–5.20). A total of 243 tibial components (99.18%) were implanted within 90 ± 3° of the MPTA.

A significant correlation was observed between the femoral component position (mLDFA) and the postoperative HKA axis (r = − 0.67, *p* < 0.001; 95%CI: − 0.73 to 0.595) as well as between the tibial component position (MPTA) and the postoperative HKA axis (r = 0.52, *p* < 0.001; 95%CI: 0.42 to 0.60).

There was a close and significant (*p* < 0.0001) correlation between intra- and interobserver measurements for all primary variables (Table 4).

**Table 4:** Intra-observer and inter-observer correlation for primary variables measured

Primary Variable Measured	Intra-observer Correlation^**$**^ (r)	Inter-observer Correlation^**#**^ (r)
Pre-operative HKA axis	0.99	1.00
Post-operative HKA axis	0.99	0.98
Pre-operative mLDFA	0.98	0.98
Post-operative mLDFA	0.97	0.97
Pre-operative MPTA	0.99	0.99
Post-operative MPTA	0.96	0.95

^$^ and ^#^: *p* value < 0.0001 for all the variables measured

## Discussion

The main findings of the present work are as follows: the HKA axis was within 180 ± 3° in 95.92% of knees, femoral components were implanted within 90 ± 3° in 97.14% of knees, and tibial components implanted within 90 ± 3° in 99.18% of knees on the coronal plane. The rate of for HKA axis and femoral and tibial component position outliers in this series was 4.08%, 2.86%, and 0.82%, respectively. There were no outliers in the valgus deformity group. There were more outliers in the > 15° varus deformity group than in the < 15° varus deformity group^.^ The number of knees with valgus deformity and varus deformity > 15° in the present study was small, so statistical significance could not be achieved for this finding. The reason for consistency in achieving the desired alignment by the described method could be that the majority of the potential errors leading to malalignment in TKA were bypassed by calculating the thickness of bone to be resected. Intraoperatively, the same thickness of bone was resected after removing cartilage and matching the thickness to that of the preoperative plan. The influence of age, sex, side, and BMI on the risk of postoperative limb malalignment and component malposition was also analyzed using the method described, and it was observed that none of these factors influenced any of the outcome variables, suggesting consistency of the method used (Table 3).

Published reports in the literature have described low rates of a neutral mechanical axis being achieved postoperatively using conventional jigs compared with CAS [17, 24, 37] and robot-assisted TKA [36, 37], while the results of PSI are mixed [20, 24, 37]. This may be due to multiple reasons. On the femoral side, Bardakos et al. [5] found that in 30 to 51% of knees, the measured VCA was > 6° or < 5°, and the VCA depended upon the neck-shaft angle (NSA) and the horizontal offset of the hip. Mullaji et al. [27] also found that the VCA was variable, ranging from 3° to 10°, and that it correlated with the severity of deformity and femoral bowing. Deakin et al. [12] compared the postoperative HKA axis in conventional jig-based TKA between two groups: group A, with a variable distal femoral VCA, and group B, with a fixed VCA. They achieved an HKA axis within 180 ± 3° in 85% of knees in group A and 69% of knees in group B; however, they did not report individual component alignment. Interestingly, despite using a variable VCA, there were 15% outliers in group A, which may be due to the discrepancy between the plan and its execution. Lampart et al. [21] prospectively collected computed tomography (CT) data from 1480 consecutive patients who underwent CT for 3D reconstruction before TKA and observed a variable femoral mechanical (FMA) angle. They concluded that due to greater variability of the FMA angle, the FMA angle seems more relevant than the hip-knee-shaft (HKS) angle in defining the strategy of realignment of the lower limb. Palanisami et al. also observed in their study that a variable VCA leads to better alignment in TKA [29]. In the present series, a variable VCA (ranging from 3° to 10°) based on the mLDFA for bone resection was used, and 13.06% of knees had a VCA different from the traditional 5–7°. Additionally, a eVCA different from the pVCA was observed in 8.16% of knees. mLDFAs within 90 ± 3° were achieved in 97.14% of knees, implying that the VCA is variable and that they further need adjustment intraoperatively to resect the planned thickness of bone. Although Kinzel et al. [19] implanted femoral components on the neutral femoral mechanical axis in 100% of 80 TKA procedures utilizing preoperative planning, they utilized CT scans for planning and postoperative evaluation. The accuracy of CT-based planning or the smaller sample size could be the reason for the better results of that study.

On the tibial side, in an attempt to improve the precision of proximal tibial resection, Magobotha et al. [25] used intraoperative fluoroscopy and achieved proximal tibial resection at 90 ± 2° on the coronal plane in 100% of a small cohort of 36 patients. Wu et al. [41] preoperatively planned proximal tibial resection on LLRs and achieved a neutral tibial implant position in 89.1% (114/128) of knees, while in the comparator group, in which they did not plan resection and depended on the extramedullary jig, they achieved a neutral tibial implant position in 79.1% of knees. The results of the current study support this trend, with a rate of tibial component position outliers of 0.82% (2/245). There were differences in the methodology of tibial resection between these two studies, which could be a reason for differences in the radiological outcomes. In the present study, a mark was aligned on the superior surface of the cutting block of the tibial jig to the line drawn from anterior to posterior along the center of the tibial spine ignoring the tibial tubercle. Wu et al. [40] measured the extent of resection from the anterior tibial margin before resection and without paring cartilage (the thickness of cartilage is variable), whereas in this study, it was measured from the center of the unaffected condyle and outer margin of the affected condyle after paring cartilage from the underlying bone before and after tibial resection to match the preoperative plan.

The reasons for the outliers observed in mechanical axis malalignment and component position in the present study were also considered. Potential reasons for these outliers include errors in the plan and its execution, differences in the center of the knee between preoperatively and postoperatively (lateral component placement causes overall varus and medial placement causes valgus), improper seating of components on one side, lifting of components during bone cement setting due to slight differences in medial and lateral ligament tension, and combinations of some or all of the above factors. Resolving these issues in future studies could potentially further reduce the number of outliers [18].

Limitations- The major limitation of this study is its single-arm design without any direct control arm for comparison with other methods, such as conventional surgery, CAS, PSI, or robotic-assisted surgery. Additionally, the number of knees in the > 15° varus deformity group and the valgus deformity group was small. All surgeries were planned and executed by a single surgeon experienced in conventional knee replacement surgery, which might have resulted in planning and execution bias unknown to the authors. Hence, we suggest that a multisurgeon and multicentric randomized controlled trial be performed for the comparison of this technique with other techniques, such as CAS and robotic-assisted TKA, to test the reliability, repeatability, and validity of this technique. The strength of this study is its prospective design and fairly large sample size.

## Conclusion

Given the favorable outcomes in terms of achieving desirable leg and component alignment in the present series, we conclude that TKA based on a modified method for preoperative planning (i.e., the true-alignment technique) described in this paper can produce reliable and consistent results without additional costs.
